# Longitudinal Analysis of Osteogenic and Angiogenic Signaling Factors in Healing Models Mimicking Atrophic and Hypertrophic Non-Unions in Rats

**DOI:** 10.1371/journal.pone.0124217

**Published:** 2015-04-24

**Authors:** Susann Minkwitz, Mirja Faßbender, Zienab Kronbach, Britt Wildemann

**Affiliations:** Julius Wolff Institute, Berlin-Brandenburg Center for Regenerative Therapies, Charité-Universitätsmedizin Berlin, Augustenburger Platz 1, Berlin, Germany; Georgia Regents University, UNITED STATES

## Abstract

Impaired bone healing can have devastating consequences for the patient. Clinically relevant animal models are necessary to understand the pathology of impaired bone healing. In this study, two impaired healing models, a hypertrophic and an atrophic non-union, were compared to physiological bone healing in rats. The aim was to provide detailed information about differences in gene expression, vascularization and histology during the healing process. The change from a closed fracture (healing control group) to an open osteotomy (hypertrophy group) led to prolonged healing with reduced mineralized bridging after 42 days. RT-PCR data revealed higher gene expression of most tested osteogenic and angiogenic factors in the hypertrophy group at day 14. After 42 days a significant reduction of gene expression was seen for Bmp4 and Bambi in this group. The inhibition of angiogenesis by Fumagillin (atrophy group) decreased the formation of new blood vessels and led to a non-healing situation with diminished chondrogenesis. RT-PCR results showed an attempt towards overcoming the early perturbance by significant up regulation of the angiogenic regulators Vegfa, Angiopoietin 2 and Fgf1 at day 7 and a further continuous increase of Fgf1, -2 and Angiopoietin 2 over time. However µCT angiograms showed incomplete recovery after 42 days. Furthermore, lower expression values were detected for the Bmps at day 14 and 21. The Bmp antagonists Dan and Twsg1 tended to be higher expressed in the atrophy group at day 42. In conclusion, the investigated animal models are suitable models to mimic human fracture healing complications and can be used for longitudinal studies. Analyzing osteogenic and angiogenic signaling patterns, clear changes in expression were identified between these three healing models, revealing the importance of a coordinated interplay of different factors to allow successful bone healing.

## Introduction

Delayed healing and non-unions are severe complications that can occur after fracture. Retrospective studies on human long bone fractures report a range between 3–15% of patients who develop non-unions [1–3], despite the advanced knowledge about bone healing processes and the continuous development of surgical interventions.

A number of cytokines and growth factors play a role in the different phases of bone healing. A dysregulation of these factors is expected to be involved in the impairment of bone healing and the formation of non-unions.

In general, non-unions can be classified into different active and inactive forms. A biologically active and vascularized non-union phenotype is defined as hypertrophic, whereas an atrophic non-union is considered to be relatively inactive and avascular [4]. This classification from 1982 still forms the basis for the definition of non-unions but has been refined several times [5, 6]. In terms of the atrophic phenotype, however, controversial findings regarding the vascularization status of these non-unions have been published. Studies investigated the amount of blood vessels in human atrophic non-unions and healing fractures and found no differences [7, 8]. In animal models with an external fixator stabilizing a bone defect with additional periosteal stripping, blood vessel formation was low after one week but increased more than in the control group after 8 weeks [9, 10]. Other animal studies clearly demonstrated, that a disturbance of angiogenesis during fracture healing leads to an avascular non-union [11, 12]. In a clinical study, arteriography was performed on the ipsilateral extremity where an open fracture with a blunt trauma occurred and they found a significantly greater incidence of delayed healing or non-union in patients with arterial occlusion [13].

It is well known that a number of risk factors influence the healing outcome. These may be either patient dependent: age, sex, drugs, smoking and nutrition state, or fracture dependent: mechanical stability, degree of soft tissue trauma, surgical interventions, infections and blood supply [14]. The search for local biological factors influencing non-union progression in patients is hindered due to the heterogeneity of fractures, presence of patient co-morbidities, and limitations in evaluation methods, which mostly evaluate endpoints. Therefore, animal models are necessary to obtain more information regarding the causes and the underlying regulatory processes of healing impairment. Several techniques have been described in literature to induce non-unions, ranging from large defects [15] to periosteum cauterization [16], induction of ischemia [17] or systemic treatment with vascularization inhibitors [11, 18]. Although, a critical size defect is a severe clinical problem, it does not address non-union formation resulting from a fracture in which also severe soft tissue damage occurs. In this study we addressed this problem by creating an open osteotomy concomitant with a soft tissue trauma to mimic a hypertrophic non-union. Disturbed vascularization is often induced by a large defect together with periosteal stripping [9, 10]. Nevertheless, the disruption of the blood supply from the outer layer of the bone leads to an increase in vessel formation and therefore does not mimic a long term disturbance of the angiogenesis. On the other side, the application of an angiogenesis inhibitor can mimic this type of dysfunction. In this study, two clinically relevant non-union models in rats mimicking a hypertrophic and an atrophic non-healing situation were used and compared to normal healing. In previous studies, dealing with the establishment of the two non-union models, a time period of 84 days was chosen to confirm the formation of non-unions. The change from a closed fracture approach (healing control group) to an open osteotomy approach already led to a pronounced delayed healing with the formation of a hypertrophic non-union after 84 days. The fractured bones gained mechanical stability similar or higher than the intact tibiae at days 42 and 84, whereas the tibiae of the osteotomy group never reached similar values [19]. At day 10, histomorphometric evaluation revealed higher values for the whole reactive callus, but lower values for the relative bone and cartilage tissue in the osteotomized tibiae. To induce an atrophic non-union the angiogenesis inhibitor Fumagillin was locally applied, which resulted in a reduction in callus formation, a disturbed revascularization and a lack of callus bridging in all animals after 84 days [20]. Both non-union models showed no healing after 84 days contrary to the fracture group where defect healing was seen after 42 days.

To get a deeper understanding about the underlying differences between these non-healing models this study pursued a comprehensive evaluation of the healing phases with regards to mineralization, vascularization and molecular conditions. Special attention was paid to important factors of the osteogenic and angiogenic signaling pathways. It is well known that both pathways are tightly linked and both play an important role in bone formation and regeneration [21]. A recent study of Kusumbe et al. [22] showed the direct connection of angiogenesis and osteogenesis. They found a new vessel type in close contact to bone lining cells, which contained endothelial cells highly positive for CD31+ and Endomucin. David et al. [23] reviewed the importance of the BMP pathway in angiogenesis. BMPs can induce angiogenesis directly or indirectly by activation of VEGF-A which mainly plays a role in the activation phase as well as in the maturation phase of angiogenesis. In addition, VEGF has been shown to regulate not only angiogenesis but also multiple biological processes in endochondral ossification and bone formation [24]. Survival and function of chondrocytes is assumed to be regulated by VEGF [25] as well as the recruitment, survival and activity of osteoclasts during the remodeling phase [26]. The balance between BMPs and their antagonists appears dysregulated in non-healing fractures [27].

Until now, no study has investigated both osteogenic and angiogenic pathways in conjunction with different clinically relevant non-healing models. In this study a closed fracture approach was used to mimic the normal healing outcome, whereas an open osteotomy led to a hypertrophic phenotype and an additional inhibition of angiogenesis to an atrophic phenotype. Gene expression analysis was performed in combination with immunohistochemistry (IHC) to increase the understanding in the factors involved in the healing process as well as to be able to describe the differences between the two models. Special attention was paid to the alterations in the callus region with respect to revascularization. The application of the polymerizing contrast agent Microfil allowed a three-dimensional analysis of the vessel network of the callus.

## Materials and Methods

### Experimental design

All studies were performed using 5 month old female Sprague-Dawley rats (Charles River, Sulzfeld, Germany) with a weight of proximately 250–290 g. A total of 180 animals were randomly divided into the three groups of 60 animals: 1. healing control group (abbreviation: control group, C), 2. hypertrophy group (abbreviation: H) and 3. atrophy group (abbreviation: A) and were further subdivided into two analysis-related groups: 1. gene expression and 2. μCT followed by histology/IHC. A healing period of 42 days was investigated with 5 different end time points after days 3, 7, 14, 21 and 42. For every time point 5–6 animals were included in the study. The animals underwent either a closed fracture of the right tibia for the normal healing (healing control group) or an open osteotomy for the hypertrophy and the atrophy group. The stabilization was carried out by an intramedullary titanium K-wire (Ø 1 mm, Synthes, Oberdorf, Switzerland) coated with the drug carrier poly(D,L-lactide) (PDLLA) (Boehringer Ingelheim, Ingelheim, Germany) following the coating protocol of Schmidmaier et al. [28] alone or additionally with the angiogenesis inhibitor Fumagillin 10% (w/w) (73 nmol Fumagillin/ K-wire; Enzo Life Science, Lörrach, Germany) [20] for the atrophy group. For mRNA analysis n = 5–6 bone samples were collected per group and time point. Six intact contralateral tibiae were used as controls. For μCT n = 5–6 animals (per group and time point) were perfused with the contrast agent Microfil MV-122 (Flow Tech Inc., Massachusetts, USA) and further histology and immunohistochemistry was performed on these specimens.

### Surgical procedure

All animal experiments were approved by the local authorities (State Office of Health and Social Affairs Berlin; Permit Number: G0305/10). The animal husbandry and surgeries were carried out in the FEM (Research Institutes for Experimental Medicine) Charité Berlin. The surgical procedures were performed as published previously [19, 20, 29]. The anesthesia was carried out by an intraperitoneal injection of a ketamine (10% 80 mg/kg body weight)/ xylazine (2% 12 mg/kg body weight) mixture. For the open osteotomy the medullary cavity of the right tibia was opened at the level of the proximal metaphysis and pre-drilled with a 0.8 mm wire. The osteotomy was set at the midshaft level using a diamond disk (Horico, Berlin, Germany). After manual fracture of the fibula the osteotomy was stabilized by an intramedullary titanium K-wire coated with PDLLA (containing Fumagillin in the atrophy group). The reposition was performed without leaving a gap. The wound was closed and treated locally with gentamycin. For the closed fracture the medullary cavity was pretreated similarly to the osteotomy procedure but the tibia and fibula were fractured in a standardized manner with a fracture apparatus [29]. The same intramedullary stabilization technique was used and the repositioning of the fracture ends was carried out without producing a gap between the bony ends. Radiographs were taken after surgery and on the euthanasia day in 2 views (posterior-anterior/ lateromedial) (Faxitron 30 kV, 10 s; Kodak DirectView CR Cassette). Euthanasia was performed in deep anesthesia by an intracardial injection of 1 mL xylazine.

### Vascular perfusion

Directly after euthanasia the abdominal cavity of the animal was opened to expose the abdominal aorta and the junction into the arteria communis of the right leg. This vessel was cannulated and was flushed first with 40 mL heparinized saline solution (100 U/mL in 0.9% NaCl) and then with the silicone rubber polymer containing lead chromate contrast agent (Microfil MV 122, Flow Tech, Massachusetts, USA). The mixing ratio of the contrast agent compounds was 5 mL diluent, 5 mL compound and 1 mL curing agent. The contrast agent was allowed to polymerize for 3 h at 4°C inside the animal, followed by fixation of the specimens in 4% Paraformaldehyde (PFA, Science Services, Munich, Germany) for 24 h at 4°C. The specimens were then stored at 4°C in neutral buffered PBS.

### Micro-computed tomography

The specimens perfused with Microfil were scanned using the Skyscan1172 high resolution micro-CT (Bruker-microCT, Kontich, Belgium) before and after decalcification of the mineralized matrix. The scan parameters were held constant for both scans with a resolution of 8.72 μm, a voltage of 60 kV, and a current of 164 μA without any filtering. For the analysis of reconstructed scans (reconstruction parameter: ring artifacts 10, beam hardening 20) the Amira software (Amira 5.7.0, Visage Imaging) was used. Image stacks of both scans were aligned using landmarks and an identical region of interest (ROI) was contoured. For volume calculations a global threshold of 2.31 g/cm^3^ calcium hydroxyapatite was used. Volume parameters for the total callus in mm^3^ and the newly formed bone (bone volume/ callus volume) in percent were extracted from the first scan. The second scan provided information of the total vessel volume (vessel volume/ callus volume) in percent, vascular connectivity in 1/mm^3^ as well as of the vessel diameter distribution. A diameter range between 15 μm and 215 μm was analyzed excluding capillaries, which have a size of around 5–8 μm in rats [30]. The first measuring span (15–35 μm) represents mainly arterioles and venules. A further classification of the vessels into small, medium, and large was made due to their diameters, neglecting the type of vessel (arteries or veins). Vessels with a diameter smaller than 55 μm were classified as small, medium-sized vessels ranged between 55–115 μm, and vessels with a diameter above 115 μm were considered to be large.

### Histology and histomorphometry

After decalcification with Ethylenediaminetetraacetic acid (EDTA, Herbelen, Berlin, Germany) for 2–3 weeks at 37°C followed by the second μCT scan, the specimens were dehydrated and embedded into paraffin. Longitudinal sections of 4 μm were made (Leica SM 2500 microtome, Wetzlar, Germany) and stained with Movat Pentachrom. With this staining differentiation of the following tissue types were possible: collagen fibers of bone = yellow-red, non-mineralized cartilage = blue-green, collagen = yellow, elastic fibers = red and nuclei = black. To quantify the area of reactive callus, zones of cartilage, woven bone, and connective tissue, image analyzing software (KS 400, Zeiss) was used as described previously [19, 29].

### Immunohistochemistry (IHC)

For IHC the slices were blocked with normal serum (goat, Vector, Biozol, Eching, Germany) and incubated overnight at 4°C with primary antibodies against Angiopoietin 2 (rabbit polyclonal antibody to ANGPT2, 1:500, Acris Antibodies GmbH, Herford, Germany) and Thrombospondin (rabbit polyclonal antibody to THBS, 1:1000, Abcam, Cambridge, UK). After incubation with the secondary antibody (anti-rabbit biotinylated, made in goat), the Avidin-Biotin-Complex (Vectastain Alkaline Phosphatase AK-5000-Kit, Vector) in combination with the Alkaline Phosphatase Substrate Kit I (Sk-5100, Vector) was used as detection system. The slices were counterstained with Methyl Green Nuclear Counterstain (Vector). A negative control was made using the secondary antibody only.

### Gene expression

The defect region (5 mm from the gap proximal and distal) without the surrounding muscles as well as the corresponding region of the intact tibiae were dissected, preserved in liquid nitrogen, and then stored at -80°C. The bone samples were pulverized with liquid nitrogen and the total RNA was isolated by the TriFast-method (Peqlab, Erlangen, Germany) in combination with a Precellys homogenizer (Peqlab). The quality and quantity of the RNA samples were assessed by Nanodrop UV/VIS- Spectrophotometer (Thermo Scientific, Waltham, USA), 1% RNA-gels and random RIN detection (Agilent Bioanalyzer, Agilent Technologies, Inc., Böblingen, Deutschland). RNA samples were stored at -80°C. Total RNA (1 μg) of every bone sample was converted into cDNA using RT^2^ First Strand Kit (Quanta Biosciences, Gaithersburg, USA). RT-PCR analyses from each bone sample were run with validated primer sets (for primer sequences see Table 1) and Sybr Green (Perfecta Sybr Green Supermix for iQ; Quanta Biosciences). The following genes were evaluated including genes of the osteogenic pathway: Bone morphogenic protein 2 (Bmp2), Bmp3, Bmp4, Bmp7, Bambi, Noggin, Chordin, Gremlin 1, Neuroblastoma 1(Dan, Nbl1), Twisted granulation 1 (Twsg1) and the angiogenic pathway: Vascular endothelial growth factor a (Vegfa), Fibroblast growth factor 1&2 (Fgf), Angiopoietin 2 (Angpt2), Angiomotin 2 (Amotl2) and Thrombospondin 2 (Thbs2). The relative expression levels of the genes of interest were calculated with the ΔΔCt method including normalization to Cyclophilin A (Ppia) and correlation to the intact bone group (n = 6).

**Table 1:** List of primer sequences.

Gene	NM_Number	Sequence forward primer	T_m_ (°C)	Sequence reverse primer	T_m_ (°C)
***Housekeeping gene***					
Ppia	NM_017101.1	gcactggtggcaagtccatct	64.8	tgctcatgccttctttcaccttc	65.0
***Osteogenic factors***					
Bmp2	NM_017178.1	gtttggcctgaagcagagac	67.2	gggacgttttcccactcatt	66.7
Bmp3	NM_017105.1	tggcggtaacacggttcgtag	65.2	tggctgtcatgggaacaacct	65.0
Bmp4	NM_012827.2	accgggcttgagtaccctga	64.2	ctctgggatgctgctgaggtt	64.1
Bmp7	NM_001191856.1	cgcagccgagttcaggatcta	65.0	cggagagctgtaagcccaggt	65.1
Bambi	NM_139082.3	tggactcactcgcaagcacag	65.0	gctgcacggaaccacagttct	64.9
Noggin	NM_012990.1	gggcatggtgtgtaagccatc	64.8	gggtggtggaactggttgga	65.4
Chordin	NM_057134	cctatgctacagcgggcacag	65.2	ccaagcccagccaatagaacc	65.0
Gremlin 1	NM_019282.3	cggcactttccttcgtgttct	63.2	tgtgctgagccttgtcaggag	64.0
Dan	NM_031609.1	aacaccttcccgcagtccac	64.9	ttcttcaggttcaggggtctgg	64.9
Twsg1	NM_001108811.1	agaaaccgtgaaccagccaca	65.1	tgtgaaaccagcggtatttgga	64.7
***Angiogenic factors***					
Vegfa	NM_001110333.1	tgcactggaccctggctttac	64.6	agggcttcatcattgcagcag	64.9
Fgf1	NM_012846	gtggatgggaccagggacag	65.1	cgcgtgcttcttggatgtgta	64.5
Fgf2	NM_019305	atcacttcgcttcccgcact	64.4	ttcgcacacactcccttgatg	64.5
Angpt2	NM_134454	ctgaccttccccaactccaca	64.6	gctgggagacaaactcgttgc	64.4
Amotl2	NM_031717.1	aggaggagctacgcaagaagca	64.9	acctgtgagggtgcctgtctg	65.0
Thbs2	NM_001169138.1	ggcagagtggaccgagtgttc	64.5	gcagagacgtatgcgggtgac	65.3

All primer pairs were designed with the Primer3web- web page and are intron spanning (exception: Noggin). Using the OligoAnalyzer 3.1 software all primer pairs were tested for self-dimers, hetero-dimers or hairpin production. The amplicon size was chosen between 150–250 base pairs. The efficiency rate of all primer pairs was above 1.8.

### Data analysis and statistics

Data of every group and time point are shown as medians and 25%- and 75% percentiles. A statistical comparison was performed between the three groups for every time point as well as for one group to the intact bone in the RT-PCR analysis using Mann- Whitney- U-Test and Bonferroni- Holm correction (PASW Statistics 18.0; SPSS, Inc.). The significance level was set to p≤0.05.

## Results

### Visualization of the healing process by x-ray and parametric evaluation of corresponding micro-CT images

**Fig 1:**
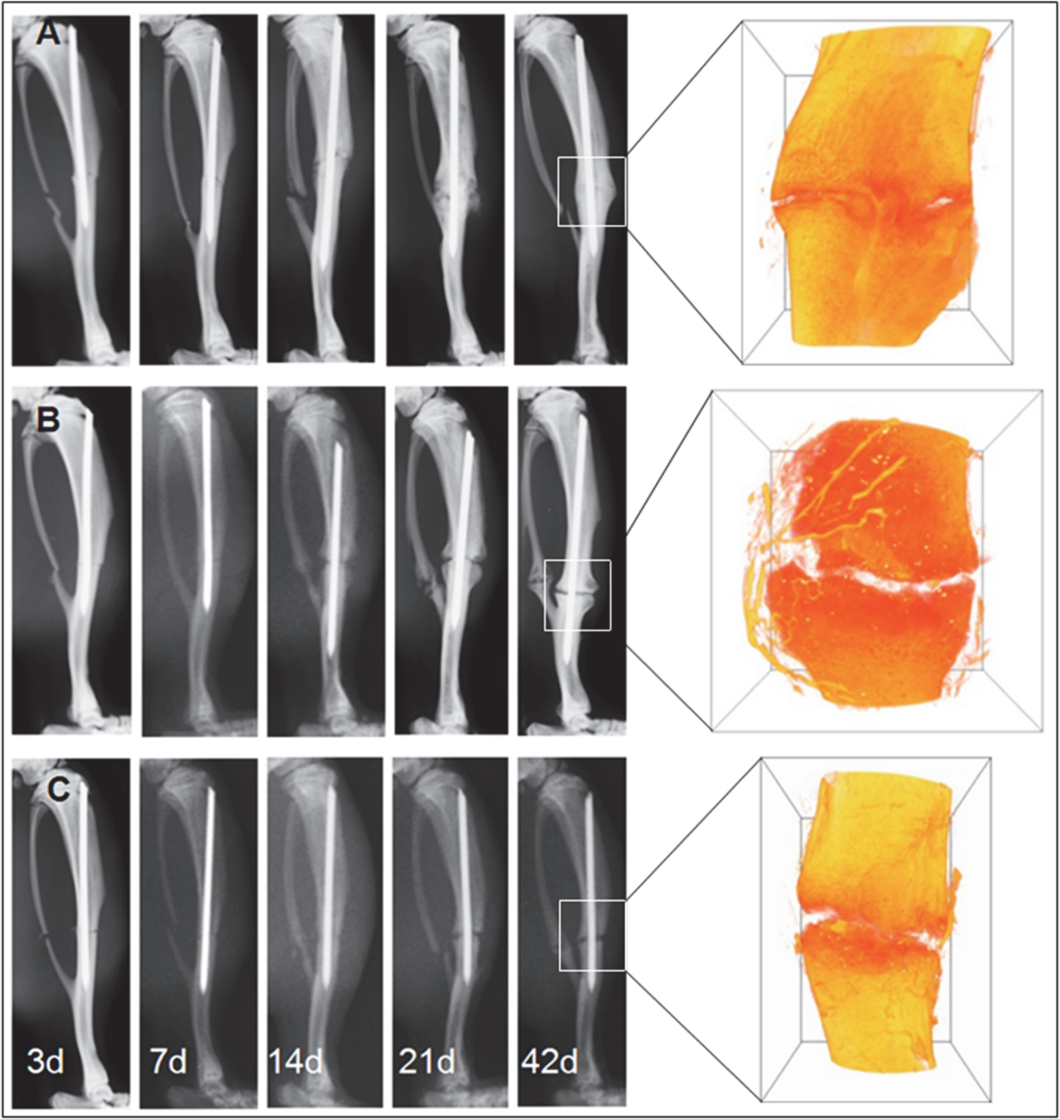
X-ray based follow-up pictures and μCT reconstructions. Radiographs are shown for day 3, 7, 14, 21 and 42 together with a 3D reconstruction image of the callus region obtained by μCT at day 42. (A) For the healing control group the right tibia of the rats was fractured and stabilized intramedullary with a PDLLA-coated k-wire. (B) Animals of the hypertrophy group underwent an open osteotomy with the same stabilization method. (C) Animals of the atrophy group were also osteotomized and obtained a local treatment with Fumagillin via the coating system of the k-wire. After 42 d post surgery periosteal bridging of the fracture gap was detectable in the healing control group but not in the other two groups.

Radiographic images showed an enhanced periosteal bridging of the fracture gap 42 days post surgery in the healing control group as compared to the osteotomy gap (hypertrophy and atrophy groups) (Fig 1). In the healing control group, the bridging callus was mineralized in 9 of 12 animals, but only in 1 from the hypertrophy and none from the atrophy group.

The total callus volume increased over time from ~45 mm^3^ at day 3 up to 207mm^3^ (174–295 mm^3^) in the healing control group and 180mm^3^ (136–260 mm^3^) in the hypertrophy group at day 14 and then decreased again. A significant reduction in the total callus volume was detectable in the atrophy group at day 14 (p(#) = 0.008 C vs A) and day 21 (p(#) = 0.009 C vs A; p(+) = 0.030 H vs A). In contrast, the bone volume fraction (bone volume/ total callus volume) increased over time and showed similar values in the three groups (**Table 2**). Only at day 7 a lower bone volume fraction was seen in the healing control group as compared to the other groups (control group: 1.2% (0.9–1.9%), hypertrophy group: 4.6% (3.3–5.6%), atrophy group: 2.6% (2.4–5.7%); p(*) = 0.010 C vs H, p(#) = 0.016 C vs A).

**Table 2:** Micro-CT data.

		Total callus volume [mm^3^]	Bone volume/ callus volume [%]
**3d**	Control group	45.9 (40.5–59.8)	0.3 (0.0–0.8)
	Hypertrophy group	41.4 (35.4–56.2)	0.0 (0.0–0.3)
	Atrophy group	42.8 (42.0–44.7)	0.0 (0.0–0.4)
**7d**	Control group	149.9 (128.4–162.6)	1.2 (0.9–1.9)
	Hypertrophy group	161.1 (118.1–186.3)	4.6 (3.3–5.6)^a^
	Atrophy group	137.0 (122.8–153.6)	2.6 (2.4–5.7)^b^
**14d**	Control group	207.4 (173.7–295.9)	10.4 (8.4–11.6)
	Hypertrophy group	180.4 (136.2–260.1)	10.1 (8.7–12.6)
	Atrophy group	151.7 (116.6–153.8)^c^	11.6 (9.4–13.3)
**21d**	Control group	157.8 (134.9–300.6)	26.3 (19.1–28.9)
	Hypertrophy group	142.7 (133.1–189.7)	17.8 (16.4–23.6)
	Atrophy group	103.0 (92.3–129.7)^d^ ^,^ ^e^	18.7 (18.5–22.3)
**42d**	Control group	135.6 (118.3–193.5)	39.5 (35.4–41.3)
	Hypertrophy group	105.3 (88.4–263.6)	37.5 (32.5–45.1)
	Atrophy group	116.3 (93.9–133.4)	34.0 (29.4–35.8)

Dimension of total callus volume [mm3] and total bone volume per callus volume [%] of all three groups over the time course obtained by μCT scans. Data are represented as medians (25%-75% percentiles). Significant differences (p≤0.05) are represented as: p(*) = C vs H; p(#) = C vs A, p(+) = H vs A; ♦ = only significant without Bonferroni Holm correction.

^a^p(*) = 0.010;

^b^p(#) = 0.016;

^c^p(#) = 0.008;

^d^p(#) = 0.009;

^e^p(+) = 0.030♦

### Vascularization

**Fig 2:**
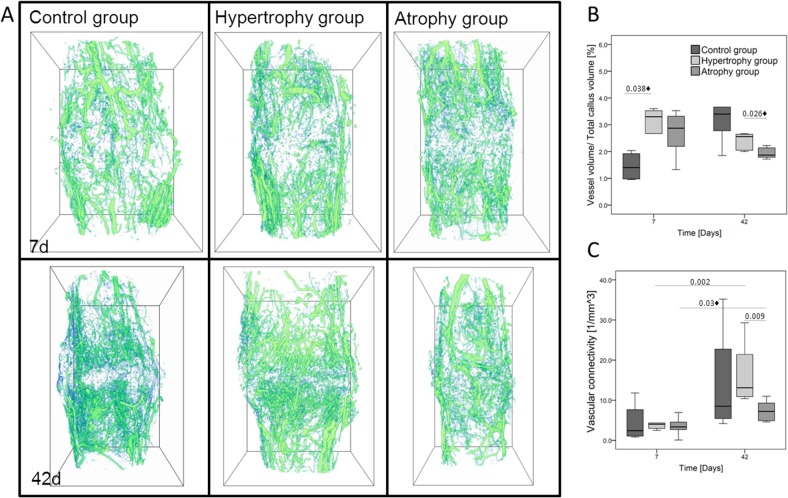
μCT angiograms of the callus regions at day 7 and 42 post surgery. (A) 3D reconstructions of the vessel networks embedded in a bounding box with a height of 8.72 mm. μCT scans were performed after decalcification of the mineralized tissue. (B) Data of vessel volume per callus volume [%] and (C) vascular connectivity (1/mm^3^). Significance level: p≤0.05; ♦ = only significant without Bonferroni Holm correction.

Vascular structures of the callus region were analyzed by μCT angiography at an early and a late time point (day 7 and 42). The vessel volume fraction (Fig 2B) in the hypertrophy and in the atrophy groups was significantly greater compared to the healing control group (control group: 1.4% (1.0–2.0%), hypertrophy group: 3.3% (2.4–3.5%), atrophy group: 2.9% (1.8–3.4%); p = 0.038♦ C vs H) at day 7. The connectivity of the vessel network (Fig 2C) was low in all groups at the early time point. The values ranged from 2.4 /mm^3^ to 4.0 /mm^3^. After 42 days an increase in the vessel volume fraction to 3.4% (2.3–4.4%) occurred in the healing control group, whereas both osteotomy groups showed a reduction in the vessel volume fraction (hypertrophy group: 2.6% (2.0–2.6%), atrophy group: 1.9% (1.8–2.2%), p = 0.052 C vs A and p = 0.026♦ H vs A). Additionally, animals of the atrophy group showed a continued low connectivity of the vessel network (control group: 8.5 /mm^3^ (4.8–29.0 /mm^3^), hypertrophy group: 13.1 /mm^3^ (10.8–23.4 /mm^3^), atrophy group: 7.2 /mm^3^ (4.8–9.7 /mm^3^).

3D reconstructions revealed a large contribution of vessels from the distal and proximal region of the callus to the total volume in the hypertrophy and atrophy group after 7 days but not in the healing control group. After 42 days a well-defined vascular network had been developed in regions of newly formed bone in the hypertrophy and healing control groups but not in the atrophy group (Fig 2A).

**Fig 3:**
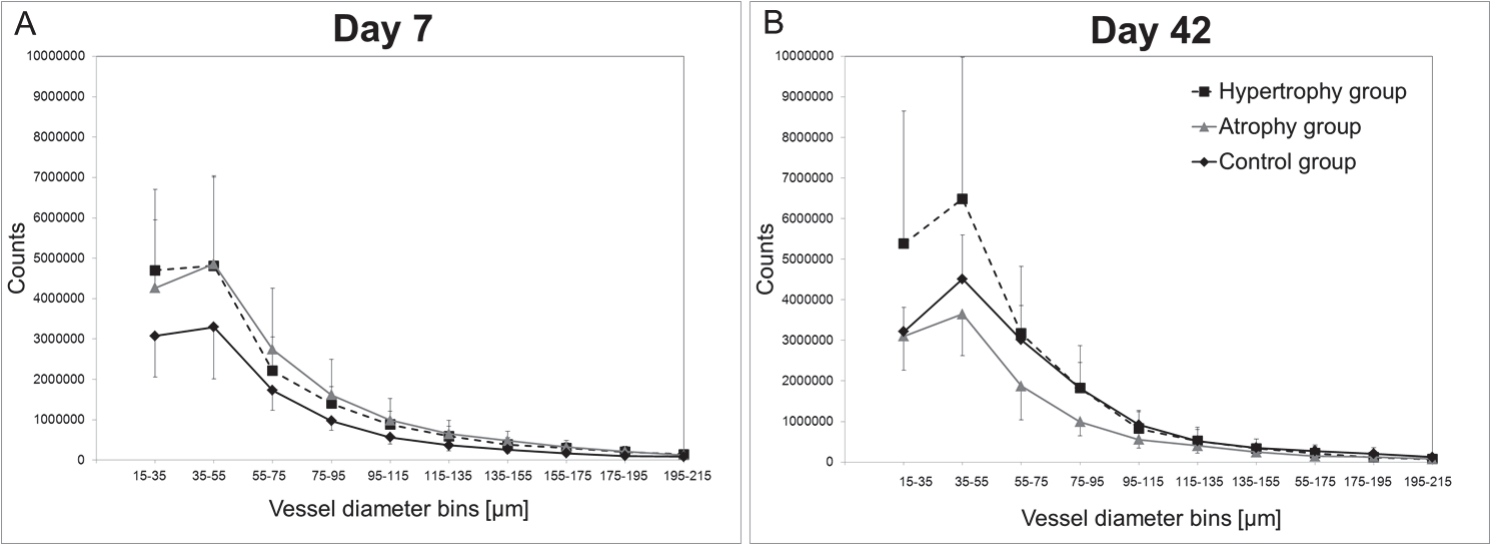
Vascular thickness histograms. Vascular thickness histograms indicating the vessel size distribution in the callus region at day 7 (A) and day 42 (B) are shown. Continuous black line = healing control group; dashed black line = hypertrophy group, continuous grey line = atrophy group. Data are represented as mean with standard deviations in one direction for better visualization. Statistical tests were performed between the groups at one time point and for one group between the two time points. Significance differences: p(C vs A) = 0.038 at day 7 in the diameter range of 75–95. In the healing control group significant differences were found between days 7 and 42: p(75–95) = 0.029. The lower amount of small vessels in the healing control group changed after 42 days, when small and medium sized vessels occurred. The local application of Fumagillin (atrophy group) decreased the formation of small and medium sized vessels.

Furthermore, the distribution of the vessel diameters in the range of 15 μm to 215 μm was analyzed (Fig 3). At day 7, the distribution pattern indicated a greater number of small vessels and slightly more medium vessels in the hypertrophy and atrophy groups as compared to the healing control group. No clear differences between the osteotomy groups were observed (Fig 3A). However, the distribution pattern changed after 42 days of healing. The calluses of the atrophy group showed fewer small and medium vessels, particularly with diameters between 35–95 μm as compared to the hypertrophy group. The vessel number of the healing control group was between both osteotomy groups (Fig 3B).

### Histology and histomorphometric evaluation

**Fig 4:**
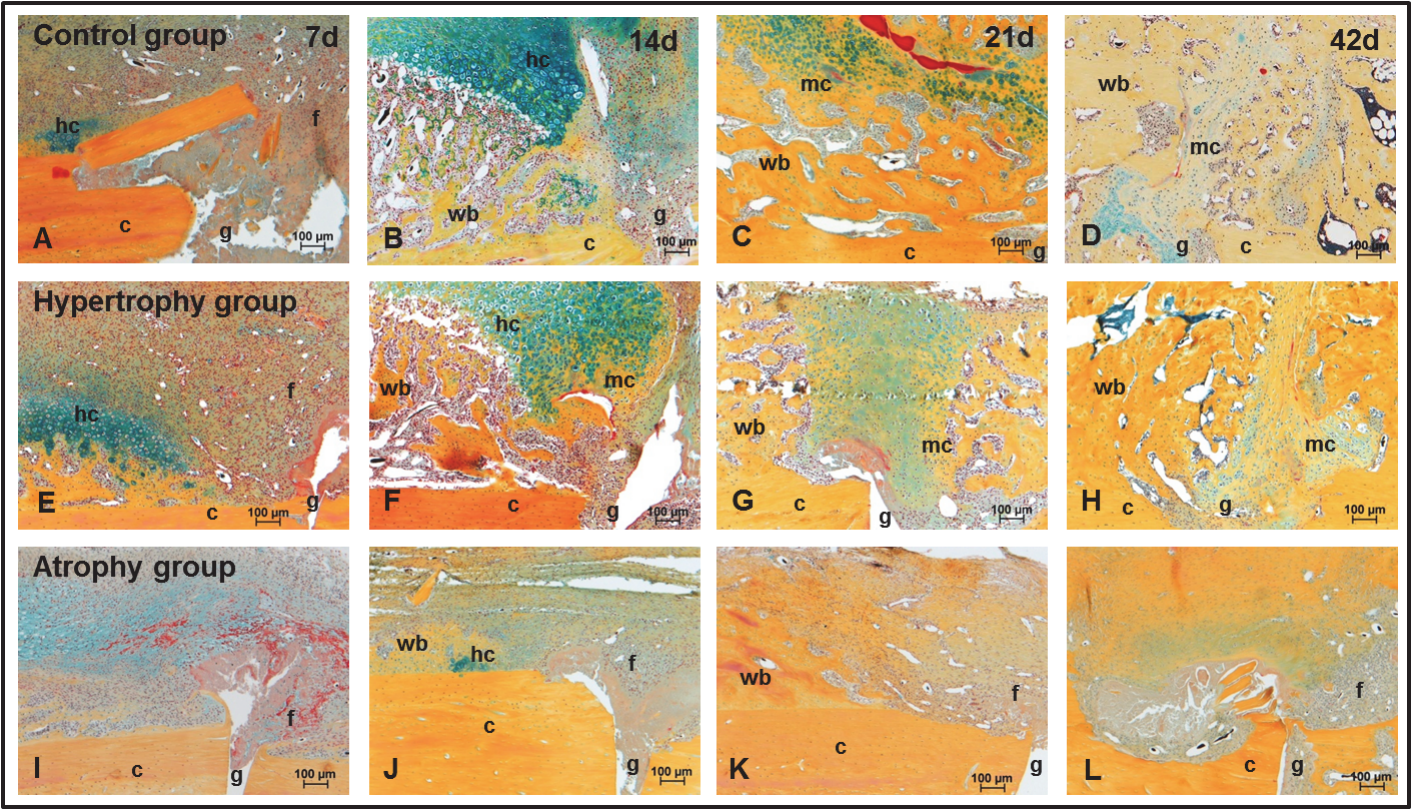
Movat Pentachrom staining. Movat Pentachrom staining of the distal/medial part of the gap region in the callus of all three groups over time. Abbreviations: c = corticalis, g = gap, hc = hypertrophic chondrocytes, wb = woven bone, mc = mineralized cartilage, f = fibrous tissue.

Based on the descriptive analysis of the periosteal callus an altered cartilage formation was detected in the atrophy group. Only small islands of hypertrophic chondrocytes could be seen over the course of time (Fig 4I–4L) and after 42 days large areas of fibrous tissue and hematoma remnants remained in the periosteal gap region of these animals. Intramembranous and endochondral ossification stages were comparable between the healing control (Fig 4A–4D) and hypertrophy groups (Fig 4E–4H). At day 42, a complete periosteal as well as intracortical bridging was seen in 3 of 6 fractured calluses, 2 were only periosteal bridged and 1 had no apparent bridging. In the hypertrophy group as well as in the atrophy group no complete bridging occurred. Mineralized bridging of the periosteal callus was only complete in 2 hypertrophic calluses but not in the atrophic calluses and intracortical bridging was not detectable at all in both groups.

**Fig 5:**
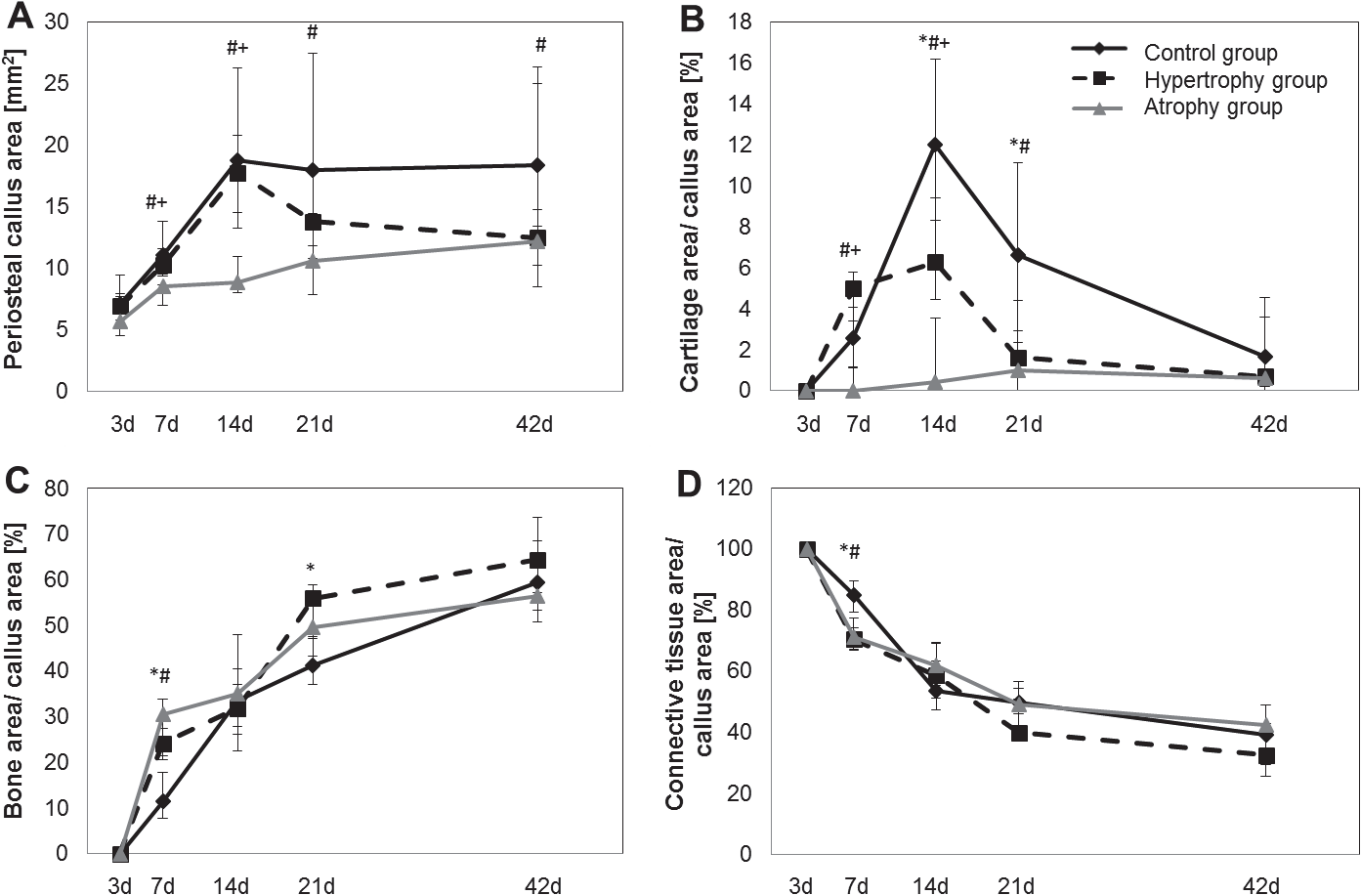
Histomorphometry data. Histomorphometry data of the total periosteal callus in mm^2^, the relative bone area, the relative cartilage area, and the relative connective tissue area in percent. Continuous black line = healing control group [C]; dashed black line = hypertrophy group [H], continuous grey line = atrophy group [A]. Data are represented as median and 25%-75% percentiles. Significance values between the groups are simplified as * = C vs H; # = C vs A, + = H vs A. Total callus area at day 7: p(#) = 0.004, p(+) = 0.004; at day 14: p(#) = 0.004, p(+) = 0.002; at day 21: p(#) = 0.015; at day 42: p(#) = 0.041♦. Bone area at day 7: p(*) = 0.004♦, p(#) = 0.017♦; at day 21: p(*) = 0.030♦. Cartilage area at day 7: p(#) = 0.017, p(+) = 0.004; day 14: p(*) = 0.030, p(#) = 0.004, p(+) = 0.008; day 21: p(*) = 0.030♦, p(#) = 0.004. Connective tissue area at day 7: p(*) = 0.004, p(#) = 0.017; ♦ = only significant without Bonferroni Holm correction.

The histomorphometric evaluation (Fig 5) confirmed a significant decrease of the total callus area between days 7 and 21 in the atrophy group as compared to the other two groups. Furthermore, the lack of cartilage formation in the atrophy group could be confirmed by measured values: day 7: 0% (0–1.1%), day 14: 0.4% (0.2–3.5%), day 21: 1% (0–2.3%) and day 42: 0.6% (0–1.7%). In the other groups an increase in the relative cartilage area occurred until day 14 to 12% (8.3–16.2%) in the healing control group and to 6.2% (4.5–9,4%) in the hypertrophy group (p(*) = 0.030 C vs H, p(#) = 0.004 C vs A, p(+) = 0.008 H vs A) followed by a decrease until day 42. Fig 5D shows the fraction of the relative connective tissue area over time, which decreased in all groups with the increase of the relative bone area. Significant differences in the relative bone area and the relative connective tissue area were detected only at day 7 (relative bone area: p(*) = 0.041♦ C vs H; p(#) = 0.017♦ C vs A; relative connective tissue area: p(*) = 0.004 C vs H; p(#) = 0.017 C vs A). At that time point woven bone formation was seen mainly in the lateral part of the callus of osteotomized tibiae whereas fractured tibiae showed less bone formation but on both sides of the periosteal callus and with more connective tissue.

### Quantitative Real Time PCR

To further investigate the different healing outcomes, as seen by μCT and histology, important factors of the osteogenic and angiogenic signaling pathways were evaluated at the RNA level. In comparison to intact bone (day 0), all genes showed regulation over the healing period.

#### Comparison between the hypertrophy and healing control groups

**Fig 6:**
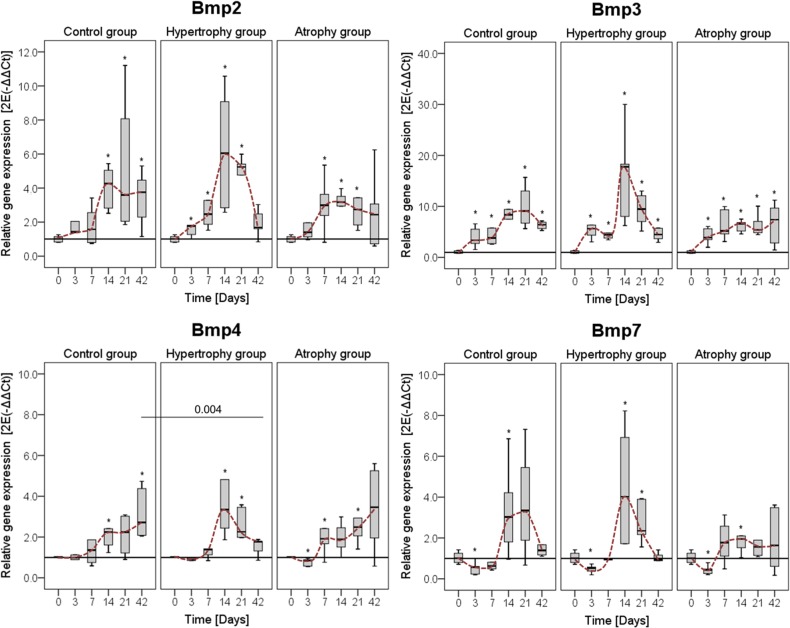
Gene expression levels of different Bmps. Gene expression levels of Bone morphogenic protein (Bmp) 2, Bmp3, Bmp4, and Bmp7 were normalized to the housekeeping gene Cyclophilin A and calculated relative to the intact bone (day 0). Comparison of the normal healing course (healing control group) to the hypertrophic and atrophic non-union for one gene in one panel. Data are presented as boxplots with median and 25%-75% percentiles. The red dashed line indicates the course of the expression values over time. Significance level: * = p≤0.05 vs. intact bone.

Up-regulation of Bmp2 and Bmp3 started at day 3 after surgery, when Bmp4 and Bmp7 showed no regulation or down-regulation at the early time points. The highest expression of the different BMPs was detectable between days 14 and 21. At these time points also the highest variability between the animals occurred. Within the tested Bmps, Bmp3 showed the highest expression values. At day 14, Bmp2, -3 and -7 tend to be higher regulated in the hypertrophy group. In contrast a significant lower expression of Bmp4 and by trend also for Bmp2 occurred in this group at day 42 (Fig 6).

**Fig 7:**
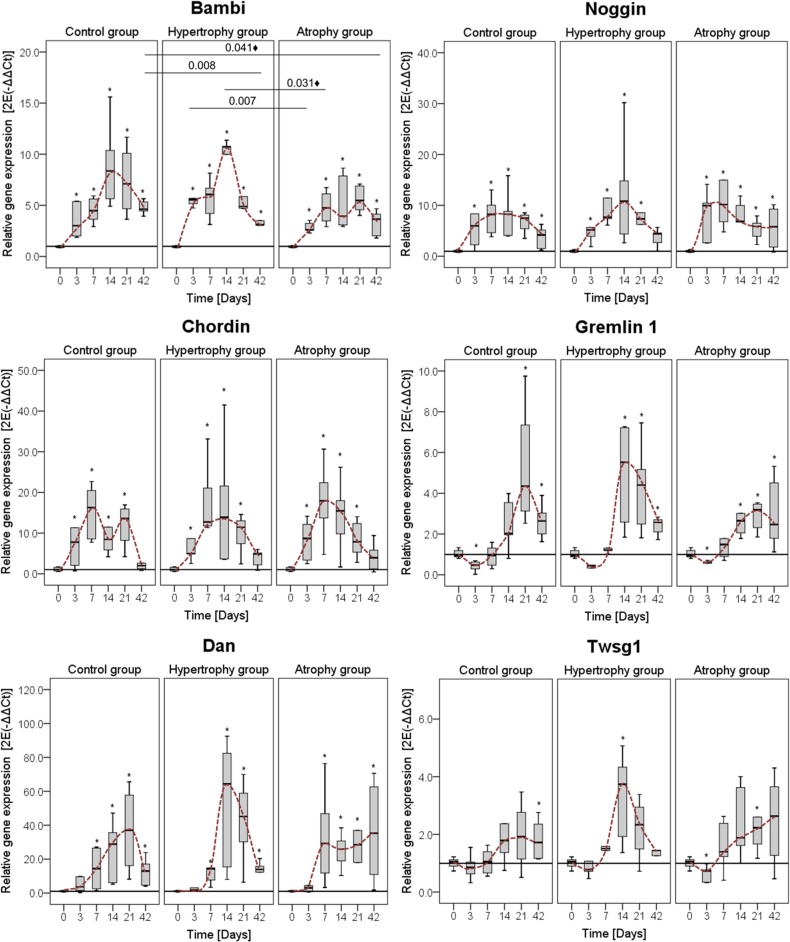
Gene expression levels of different Bmp antagonists. Comparison of mRNA levels of the three groups for the Bmp antagonists: Bambi, Noggin, Chordin, Gremlin 1, Neuroblastoma 1 (Dan) and Twisted granulation 1 (Twsg1). Data were normalized to the housekeeping gene Cyclophilin A and calculated relative to the intact bone (day 0). Comparison of the normal healing (healing control group) to the hypertrophic and atrophic non-union for one gene in one panel. Data are presented as boxplots with median and 25%-75% percentiles. The red dashed line indicates the course of the expression values over time. Significance level: * = p≤0.05 vs. intact bone; ♦ = only significant without Bonferroni Holm correction.

The analysis of the Bmp antagonists revealed very high expression levels of Dan but also of Chordin and Noggin during bone healing. Bambi, Noggin, Chordin and Dan were up-regulated already at day 3, whereas Gremlin 1 and Twsg1 started to be up-regulated at later time points in all groups. In general, most Bmp antagonists had their highest expression, similar to the Bmps, between days 14 and 21. As an exception to this expression pattern, Noggin and Chordin show highest values between day 7 and 14. Noggin, which is known to block Bmp2, -4 and -7 activity, showed similar expression pattern in both groups over time. As seen for the Bmps, calluses in the hypertrophy group tended to have higher expression values for the tested antagonists at day 14 compared to the healing control group. Within the healing control group, Gremlin 1, Dan and Twsg1 showed maximal expression at day 21. At the end of the healing process a significant down-regulation of Bambi was detectable in the hypertrophy group (Fig 7).

**Fig 8:**
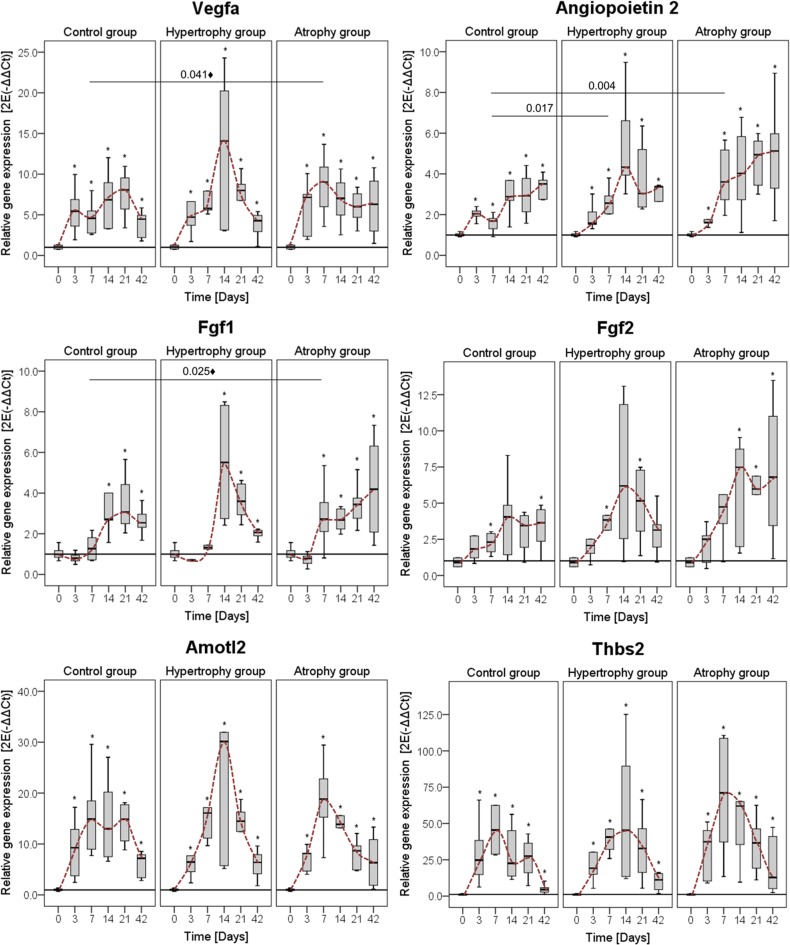
Gene expression levels of different angiogenic factors. The pro-angiogenic factors: Vascular endothelial factor alpha (Vegfa), Fibroblast growth factor (Fgf) 1 & 2 and Angiopoietin 1 & 2 as well as the anti- angiogenic factors: Angiomotin (Amotl) 2 and Thrombospondin (Thbs) 2 were tested. Data were normalized to the housekeeping gene Cyclophilin A, further calculated relative to the intact bone (day 0) and presented as boxplots with median and 25%-75% percentiles. The red dashed line indicates the course of the expression values over time. Significance level: * = p≤0.05 vs. intact bone; ♦ = only significant without Bonferroni Holm correction.

For the angiogenesis markers, Vegfa showed highest expression among the proangiogenic factors. Both angiogenesis inhibitors (THBS2 and Amotl2) were highly up-regulated during the healing process. The main difference between the hypertrophy and healing control groups was seen again at day 14 with higher expression levels in the hypertrophy group (Fig 8).

#### Comparison between the atrophy and healing control groups

Expression levels of BMPs tended to be higher at day 7 in animals of the atrophy group compared to the healing control group but showed only marginal changes over the later time points (Fig 6). The expression of Bambi, Noggin and Chordin was comparable between both groups, whereas Dan and Twsg1 stayed high in the atrophy group as opposed to the healing control group, where it decreased at day 42 (Fig 7).

The angiogenic factors Vegfa, Fgf1 and Angiopoietin 2 were significantly up-regulated at day 7 in the atrophy group. Furthermore, Fgf1, -2 and Angiopoietin 2, but not Vegfa, showed a continuous increase over time. The anti-angiogenic genes Angiomotin 2 and Thrombospondin 2 showed a trend towards a higher expression value in the atrophy group at day 7 (Fig 8).

### Immunohistochemistry

Angiopoietin 2 (ANGPT2) as a pro-angiogenic factor and Thrombospondin (THBS) as an anti-angiogenic factor were further analyzed with immunohistochemistry. In general, both staining reactions showed only weak signals but could clearly be differentiated from the lack of signal in the negative control (secondary antibody only, data not shown).

**Fig 9:**
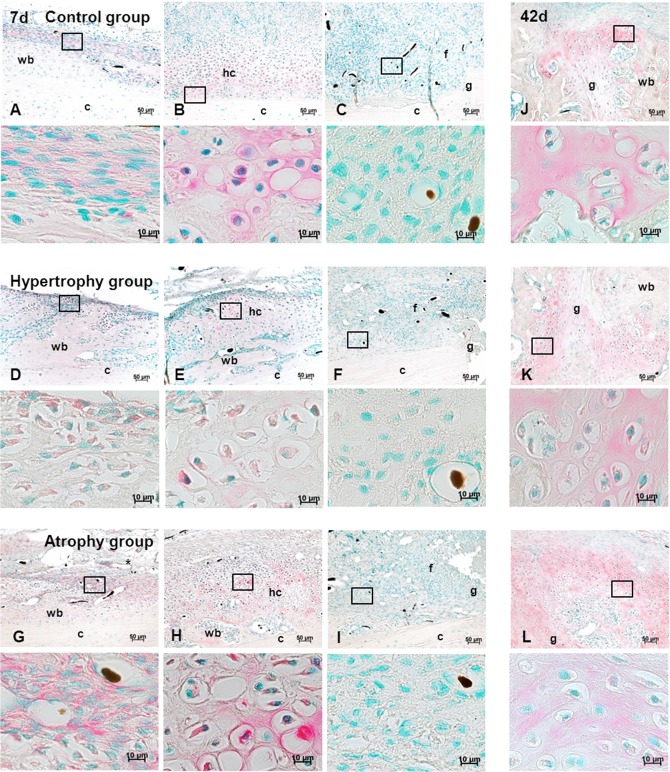
Anti-Angiopoietin 2 staining. Immunohistochemical anti-Angiopoietin 2 staining (red) of sagittal sections of all three groups at day 7 (A-I) and 42 (J-L) in combination with a nuclear counter staining (green). Higher magnification images are presented under the corresponding overview image and the region is indicated by a black square. Images of column 1 (A, D, G) show the cells lining the newly formed bone, column 2 (B, E, H) show staining of areas with hypertrophic chondrocytes. Column 3 (C, F, I) as well as column 4 (J, K, L) show the gap region after 7 and 42 days, respectively. Scale bar = 50 μm and 10 μm. Abbreviations: c = corticalis, g = gap, hc = hypertrophic chondrocytes, wb = woven bone, mc = mineralized cartilage, f = fibrous tissue. Back dots = Microfil remnants in the vasculature.

During the early healing phase ANGPT2 was mainly produced by cells lining the newly formed bone at a distance from the fracture/ osteotomy gap (Fig 9A,9D and 9G), by endothelial cells of larger vessels (Fig 9G, *) and maturing chondrocytes (Fig 9B,9E and 9H). At that time point no difference in signal intensity could be detected between the groups. After 42 days, mainly mature and hypertrophic chondrocytes secreted ANGPT2 near the fracture/ osteotomy side. Calluses of the healing control and hypertrophy groups which showed nearly complete periosteal bridging harbored few positive cells on the periosteal edge or near the corticalis gap (Fig 9J and 9K). In calluses of the atrophy group secretion of ANGPT2 was quite heterogeneous. Some specimens showed ANGPT2 secreting chondrocytes (Fig 9L), whereas others with mainly fibrous tissue in the middle of the callus showed only ANGPT2 positive cells in the osteoid surrounding the woven bone. In all calluses of the atrophy group, the ANGPT2 signal was more intense in cells of the callus periphery compared to the other two groups.

**Fig 10:**
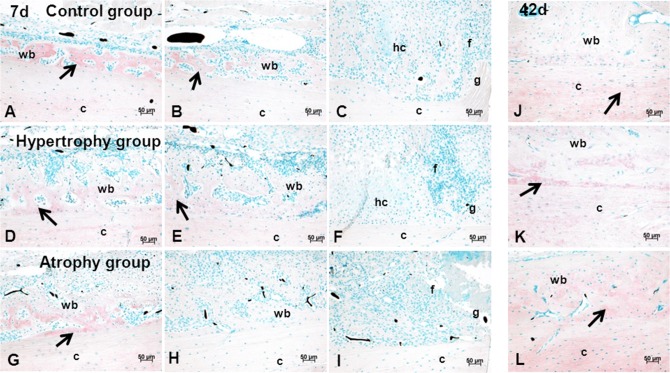
Anti-Thrombospondin staining. Immunohistochemical anti-Thrombospondin staining (red) of sagittal sections of all three groups at day 7 (A-I) and 42 (J-L) in combination with a nuclear counter staining (green). Images of column 1 (A, D, G) shows areas of woven bone at the periphery, column 2 (B, E, H) shows the transition of stained and unstained areas in the middle part of the distal/medial callus and column 3 (C, F, I) shows the gap region after 7 days. After 42 days (J, K, L) THBS was enriched not only in the newly formed bone but also in the corticalis. Scale bar = 50 μm. Abbreviations: c = corticalis, g = gap, hc = hypertrophic chondrocytes, wb = woven bone, mc = mineralized cartilage, f = fibrous tissue. Arrow = THBS positive areas; Back dots = Microfil remnants in the vasculature.

THBS could be located as a secreted protein linked to the extracellular matrix (ECM) of woven bone but also lamellar bone. Only osteoblasts and osteocytes, but not endothelial cells, fibroblasts, or chondrocytes, could be detected as cells secreting THBS. At day 7, the staining signal was most intense in the woven bone at the periphery of the callus and decreased towards the fracture/osteotomy gap (Fig 10A–10I). No differences in the signal intensity or dispersion between the three groups were detectable. At day 42, THBS was not only present in the ECM of the woven bone but also in the lamellar bone of the corticalis. Notably, in the periphery the signal was highest in the woven bone structures whereas near the gap mainly the corticalis, which undergo remodeling, showed THBS expression (Fig 10J–10L). Atrophic calluses showed higher signal intensity compared to the other two groups at day 42.

## Discussion

The most common long bone lesions are tibia fractures and depending on the anatomical location and the severity of trauma, they can have the highest non-union rate [31]. Bone healing processes are well characterized but the question remains as to what happens particularly when patients develop impaired bone healing or a non-union, especially since bone has such a remarkable capacity to regenerate. In an attempt to answer this question, three different clinically relevant healing models in rats, mimicking normal healing but also hypertrophic and atrophic non-union, were analyzed.

Based on radiographic images, μCT and histology, two important differences were observed in the healing processes in the healing control group versus the hypertrophy group. First, hypervascularization was detected in hypertrophic calluses at the beginning of the healing cascade. Second, a lack of bony intracortical callus formation and partially periosteal bridging occurred in these calluses.

It is known that the development of a hypertrophic callus in animal models is mainly caused be instability of the fixation method [32] and is accompanied by enhanced cartilage formation. In this study all tibiae were stabilized in the same manner and the fibulae were broken manually to guarantee the same degree of stability. Histomorphometry data revealed higher values of cartilage area in the hypertrophy group at day 7 but lower values at day 14 and 21 compared to the healing control group. The phenomenon at the later time points could be explained by small bone fragments (bending wedge) that form during the fracture process (healing control group) stimulating cartilage formation at different parts of the callus. A smooth cortical surface, as produced by the osteotomy, provides less stability than the irregular cortical surface after fracture and may account for the early cartilage formation in the hypertrophy group.

The vascularization of the callus region was assessed by μCT 3D-imaging. At the early healing time point hypertrophic calluses showed a higher blood vessel volume per total callus volume compared to the healing control group. This enrichment of vessel volume was caused by a high number of microvessels with small and medium diameters mainly located in the peripheral parts of the periosteal callus, but not in the direct gap region. It is well known that the surrounding soft tissue significantly contributes to the blood supply of the fractured bone immediately after the injury and persists until the normal blood circulation is restored [33]. Taking the different surgical techniques into account the blood supply from an intact connection to the surrounding muscle, like seen after a closed fracture, might stimulate the early fracture healing without the need of extended vessel ingrowth. In case of the osteotomy approach early vessel ingrowth is needed due to the reduced blood supply from the surrounding tissue. After 42 days the architecture of the vessel network changed towards a well-defined network inside the newly formed bone in both groups. In this network an increase in vessels with a medium-sized diameter and an increase in vessel connectivity in both groups were found. The hypervascularization had therefore no direct negative effect on the later development of the vessel network inside the bone structures, but might be an indicator for delayed healing processes.

At the mRNA level, no differences in expression levels of angiogenic factors occurred at the first two time points (days 3 and 7), although a clear difference in vessel network formation was detectable by angiography between both groups. A higher expression of osteogenic but also angiogenic genes was detected at day 14 in the hypertrophy group indicating enhanced healing processes at the osteotomy gap. This might be linked to an enhanced availability of progenitor cells resulting from the improved blood supply. In contrast, in the healing control group the highest expression of most investigated factors such as Bmp3, Bmp7, Gremlin 1, Dan, Twsg1 as well as Vegfa, Fgf1 and Amotl2 was seen at day 21. At that time point resorption of calcified cartilage and osteoblastic recruitment is induced [34]. At the end of the analyzed healing period significantly less Bmp4 was expressed in the hypertrophy group as well as Bambi and also a downwards trend of Bmp2 and Twsg1 expression was observed. Bmp4 is a factor supporting the later stages of fracture healing and associated to long bone fracture non-unions in patients [35]. Taking into account that the healing process occurs by a well-balanced interplay of a multitude of factors stimulating, antagonizing, or compensating each other, it can be speculated that these changes in expression can be sufficient to delay healing.

In general, a variety of different scenarios were found in human studies on non-unions. For example, lower protein synthesis of BMPs with comparable BMP antagonists was detected in human non-union biopsies when compared to fracture calluses [27]. On the contrary, another study by Fajardo et al. [36] measured higher expression levels of Bmps as well as of the Bmp antagonists in non-unions than in normal healing situations. A third study observed no differences in Bmp expression in delayed healing versus non unions [37]. The limitation of all these studies was the single time point analysis and the heterogeneous patient cohort. Osteotomies in mice stabilized with a non-rigid fixator showed enhanced expression of wnt- and BMP-molecules and their antagonists when compared to a rigid fixation [38]. The study of Lienau et al. [39] applied an unstable fixation method to induce impaired bone healing in sheep and found different temporal expression patterns of genes regulating blood vessel formation. Both studies worked with an unstable fixation technique to induce delayed healing, which was not the case in our study.

When comparing the normal healing to the atrophic non-union model the local application of Fumagillin at the early stage of bone healing had not only a negative effect on the long term vascular network formation but also on chondrogenesis. A slight reduction of the vessel volume could already be seen at the early time point when compared to the hypertrophy group. After 42 days, a significantly lower vessel volume and a lack in connectivity of the vessel network had been manifested in the atrophy group. Comparing the late stage of healing to the early one in this group, the vascular thickness histograms showed a reduction in the number of small and medium sized vessels.

Additionally, although the atrophic calluses showed a reduction of the reactive callus size, the periosteal woven bone formation was not influenced. But the chondrogenesis was highly altered. The lack of cartilage development in the atrophy group after 42 days was associated with residual hematoma and formation of fibrous tissue in the gap near regions of the callus. A comparable tissue composition was seen in the study of Hausman et al. [11] typically in clinical non-unions failing to undergo chondrogenesis.

Based on previous *in vitro* release kinetic studies [28, 40, 41], a burst release of the angiogenesis inhibitor during the first hours followed by slow release over several days was expected in our model. Fumagillin would thereby have a high influence on the biological processes at the beginning of the healing cascade and in particular endosteal, intracortical and near the gap region but to a lesser extent in the periosteal periphery. This would explain the similar hypervascularization in the periphery of the periosteal callus as seen in the hypertrophy group at day 7. In contrast, around the gap, Fumagillin diffusion into the surrounding tissue caused a later disturbance of the vessel network formation in combination with a lack of cartilage formation. It is therefore not a model mimicking periosteal injuries but rater complications occurring during the fracture healing period. Fumagillin is naturally produced by Aspergillus fumigatus having an inhibitory effect on the methionine aminopeptidase type 2 (MetAP2) and downstream processes of the non-canonical Wnt signaling pathway [42–44]. Wang et al. [45] demonstrated that TNP470 (Fumagillin derivate) leads primarily to a cell cycle arrest in endothelial cells (ECs) but does not affect other non-endothelial cells types. Furthermore we have shown that human osteoblast like cells (POBs) are not influenced by this agent [20]. We therefore assume that the changes seen in the atrophy group were associated to altered EC behavior followed by a disturbed vessel formation.

RT-PCR results of atrophic calluses showed an attempt of the organism to reverse the inhibition of angiogenesis by significant up-regulation of angiogenic factors such as Vegfa, Angiopoietin 2 and Fgf1 and by trend also Fgf2 starting at day 7 with a continuous increase over time. Angpt2 is associated with formation of larger vessels and branching of existing vessels [34]. The up-regulation of this factor indicates a high contribution of existing vessel structures from the periosteal region to the blood supply of the callus. This goes along with the results of the vessel thickness histogram where mainly small vessels were absent and the 3D vessel network showed only larger connective vessels in the periphery of the callus but not near the gap. Interestingly, Vegfa expression was only significantly enhanced at day 7 showing no further up-regulation over time. It is known that under hypoxic conditions, occurring during hematoma formation, and later on during cartilage resorption and bone remodeling, Vefga is expressed [46]. The increased expression at day 7 might therefore be linked to a delay in hematoma degradation in the atrophy group. The lack of Vegfa during the later time points might be associated with the absence of cartilage formation and a reduction in activity of endothelial cells induced by Fumagillin treatment. In tumorgenicity studies a direct inhibition of VEGF secretion of tumor cells after TNP-470 treatment led to an indirect effect on the proliferation and activity of ECs [47]. Direct blockage of VEGF production in cells such as chondrocytes or osteocytes was so far not investigated.

Furthermore, anti-angiogenic regulators such as Angiomotin like 2 (Amotl2) and Thrombospondin 2 (Thbs2) were highly expressed in the atrophy group at day 7 and slightly higher at day 42 compared to the control group. At the protein level, an enhanced presence of THBS could be detected in the atrophic calluses at day 42, but no clear differences at day 7. Likewise, higher protein synthesis at day 42 was seen for the pro-angiogenic factor ANGPT2. ECs, osteoblast precursors as well as maturing and hypertrophic chondrocytes are sources for ANGPT2 synthesis [34, 48]. Similar to THBS, occurrence of this protein shifted from the periphery of the callus region at day 7 to parts near the gap after 42 days.

The analyzed osteogenic factors (Bmp2, -3, -4 and -7) showed also a dysregulated expression profile in the atrophy group over time. The up-regulation at day 14 and 21 as seen in the hypertrophy and the healing control groups could not be detected in atrophic calluses. Bmp antagonists such as Gremlin 1 and Dan showed similar down-regulation at that time. Notably, Bmp4, Dan, Twsg1, and to a lesser extent Gremlin 1 had higher expression levels in the atrophy group at day 42. Other than Dan, all of the up-regulated factors are known regulators of angiogenesis by effecting EC behavior [34, 49, 50]. A similar general down-regulation of Bmps but also of their antagonists was demonstrated by Niikura et al. [16], who compared closed femoral fractures with and without periosteal cauterization in rats. Interestingly, the same down-regulation of osteogenic factors together with an up-regulation of angiogenic factors resulting in a delayed healing was demonstrated in osteotomized animals treated with nicotine [51]. It is well known that BMPs trigger the formation of bone and cartilage [34] and that MSCs that differentiate into the chondrogenic lineage secrete BMPs such as BMP2 and BMP4 [52]. It was also described that BMP7 better than BMP2 is able to stimulate chondrogenic differentiation in adipose tissue-derived MSCs [53]. The lack of chondrogenesis in our atrophy model might therefore be associated with the lower expression levels of osteogenic genes at day 14 and 21.

As outlined before, studies on human fracture healing are highly limited and cannot produce standardized and time dependent data of the healing process. Especially, information about the changes in tissue composition over time are rare and the source for this knowledge is mainly based on animal studies. Nevertheless, animal models are limited by the manipulation necessary to induce a specific phenotype of healing and may therefore not reflect the exact healing situation as seen in the clinic. In this study, the open osteotomy in the hypertrophy model mimics an open fracture with a soft tissue trauma which can occur due to accidents. The application of the angiogenesis inhibitor Fumagillin is in fact a more artificial intervention but the influence of Fumagillin is restricted to EC proliferation.

The surgical intervention in the healing control group is different to the other groups and is meant to mimic normal healing without soft tissue trauma, infection or disturbed angiogenesis but not as a control for the models per se. Using a closed fracture in combination with the angiogenesis inhibitor might also lead to the development of an atrophic non-union but this has to be proven by establishing an additional model.

Another methodical limitation of this study was the application of the contrast agent Microfil. The quality of the perfusion was assessed macroscopically by the yellow staining of larger vessels of the muscle and a uniform yellow coloration of the right hind paw. Unperfused vessels cannot be visualized by μCT and a measure of accuracy of the perfusion procedure was not possible. Nevertheless, it is a very promising tool to visualize the vasculature as a 3D-network inside the bone or mature vessels. However, we observed small islands of contrast agent in the early callus but with no clearly connected vessel network. Furthermore, EC and smooth muscle cells of the vessels were destroyed in many cases and we assume that this happened due to the osmotic pressure of the contrast agent. This might be a problem for immature vessels to sustain the pressure resulting in disruption of the vessels. In addition, the visualization of capillaries was not possible due to the viscosity of the contrast agent necessary to detect it with μCT and the resolution and scanning time necessary to scan the total callus part.

## Conclusion

In conclusion, we demonstrated that the change from a closed fracture to an open osteotomy approach already leads to a prolonged healing cascade with an impaired periosteal bridging after 42 days in a rat bone healing model. RT-PCR data revealed changes starting at day 14 with higher expression of osteogenic and angiogenic genes in the hypertrophy group. A further local application of the angiogenesis inhibitor Fumagillin at the beginning of the healing cascade fosters a negative healing outcome leading to a reduction in callus formation and a disturbed revascularization. A significantly higher expression of angiogenic genes at day 7 and an increase over the later time points could be detected in the atrophy group. Osteogenic genes were less up-regulated as compared to the other groups.

The present study contributes to a deeper understanding of the molecular processes which are initiated after disturbance of normal bone healing either by a larger soft tissue trauma or inhibition of the angiogenesis. The results show that the healing is regulated by a balanced expression of various factors inducing bone and vessel formation and that a misbalance can be seen in the two impaired healing models. To get a deeper understanding of the various processes that are influenced in the different models microarray analysis is planned.
